# Birth Control

**Published:** 1920-07-31

**Authors:** 


### Birth Control.

### THE QUESTION OF OVER-POPULATION.

The subject of birth control, like that of the prevention of venereal disease, sharply divides publicists on the grounds of morality, and birth control is further complicated by considerations of national preservation and protection. It is generally admitted that birth control is now widely practised, so it is perhaps a bit late in the day to bother about whether it is to be deplored, for no pious pronouncements against it will exercise any effective influence. The more practical policy—whether the practice is to be deplored or not—is to direct it on to the best lines, since it admittedly exists and cannot be prevented. We feel that opinion will sooner or later come round to the view that venereal prevention must be faced in the same practical way. Promiscuous intercourse exists and will exist, deplore it as you may; therefore do the best to mitigate its evil possibilities. However, even if those basic propositions of principle were generally accepted—which is by no means the case—the methods of procedure would still be acutely controversial.

The strongest public advocacy of the principle of birth control that we have read for some time was the paper by Dr. C. Killick Millard (Medical Officer of Health, Leicester), at the Birmingham Health Congress, though the subsequent discussion revealed all the existing divergencies of opinion on the matter. He started upon two facts—first, the remarkable tendency to birth-rate decline in the last forty years (he regards the present substantial recovery as a purely temporary phenomenon), and second, the practice of voluntary limitation of families by almost all sections of the community except the lowest. Decline of birth-rate does not mean decline in population, because there is a difference between the percentage rate per population and the actual increase. For instance, from 1901 to 1911, the population of England and Wales increased by 3,543,000, despite the falling birth-rate. A striking fact is that simultaneously with the decline in the birth-rate, there has been an almost equally remarkable fall in the death-rate, and especially in the infant death-rate. Similar phenomena have occurred in France, Belgium, Holland, and Germany. Dr. Millard foreshadows the aggravation of the problem of the world's food supply—especially in an island like ours—of the progress towards the definite end of the world's coal supply, and the international strife caused by pressure of population.

### A Terrible Computation.

If the increase of population continues, there must come an inevitable time of over-population. Between 1801 and 1911, the population of England and Wales quadrupled. Assuming that the same rate of increase were maintained for another 330 years, we should have the utterly preposterous figure of 2,300,000,000—greater than the present entire population of the globe; the same thing would have been happening in every country in the world. Asia has already reached the point of "saturation" on population. Upon every consideration Dr. Millard welcomes the decline in the birth-rate, and hopes that better education will overcome the present dysgenic effect of the falling rate.

On the ethical aspect of the question, Dr. Millard notes with satisfaction that high ecclesiastical authority is not insensible to the force of reason and logic, and some distinguished representatives of the church have had the courage to take up an independent line. He quotes Dean Inge, and Dr. Russell Wakefield, and he declares finally that it is quality, not quantity of population that any nation really needs; to obtain quality, we must be prepared to sacrifice quantity. He dismisses entirely the "so-called patriotic" view of the birth-rate problem. He points out that the territories of the British Empire are now about the only ones remaining suitable for permanent settlement by Europeans. Emigration on a larger scale would only postpone the evil day. When those territories are filled up, "nothing but birth control on a much greater scale than we have experienced at present can save Europe from further devastating wars or a great increase in mortality." Is it better (he asks, in effect) to have scientific control or to leave the matter to Nature's brutally inexorable laws?

